# Green synthesis of silver nanoparticles using Sudanese *Candida parapsilosis*: a sustainable approach to combat antimicrobial resistance

**DOI:** 10.1186/s12866-025-04038-9

**Published:** 2025-05-21

**Authors:** Nesreen A. A. Ibrahim, Humodi A. Saeed, Samar M. Saeed, Osama Mohamed, Omnia H. Suliman, Sabah A. E. Ibrahim, Sofia B. Mohamed

**Affiliations:** 1 Department of Microbiology, Faculty of Medical Laboratory Sciences, Sudan international university, Khartoum, Sudan; 2 Department of Microbiology, Faculty of Medical Laboratory Sciences, Sudan University of Science and Technology, Khartoum, Sudan; 3 Department of Microbiology, College of Medical Laboratory Sciences, Sudan University of Science and Technology, Khartoum, Sudan; 4 Department of Molecular Biology, National University Biomedical Research Institute, National University-Sudan, Khartoum, Sudan; 5 Department of Medicine & Surgery, Dubai Medical University, Dubai, UAE; 6 Bioinformatics and Biostatistics Department, National University Biomedical Research Institute, National University, Khartoum, Sudan; 7 Malaysia Department of Applied Physics, Faculty of Science and Technology, Universiti Kebangsaan Malaysia, Bangi, 43600 UKM Selangor Malaysia

**Keywords:** *Candida parapsilosis*, Green synthesis, Silver nanoparticles, Antibacterial activity, membrane integrity

## Abstract

**Background:**

Antimicrobial resistance (AMR) is a critical global health challenge, particularly in Sudan, where the overuse and misuse of antibiotics have driven the rise of multidrug-resistant (MDR) pathogens. Conventional antimicrobial strategies often fall short due to rapid resistance development and limited efficacy, highlighting the need for novel approaches. Nanotechnology offers promising alternatives, with silver nanoparticles (AgNPs) demonstrating potent broad-spectrum antimicrobial activity. This study aims to develop an eco-friendly synthesis of AgNPs using *Candida parapsilosis* (*C. parapsilosis*), an untapped yeast strain isolated from Sudanese soil, to combat AMR.

**Results:**

Biosynthesis of AgNPs using *C. parapsilosis* was successfully confirmed through UV-Vis spectroscopy, X-ray diffraction (XRD), and high-resolution transmission electron microscopy (HRTEM), revealing well-defined nanoparticles. The biosynthesized AgNPs exhibited strong antibacterial activity against both ATCC reference strains and MDR clinical isolates of Gram-positive and Gram-negative bacteria, with inhibition zones increasing in a concentration-dependent manner. At optimal concentrations, inhibition zones reached 29 mm for *Pseudomonas aeruginosa (P.aeruginosa)* (ATCC 27853), while clinical isolates of *Salmonella typhi* (*S. typhi*) (24.5 ± 0.58 mm) and *Escherichia coli* (*E. coli*) (23.8 ± 0.79 mm) exhibited significant susceptibility. Minimum inhibitory concentration (MIC) and minimum bactericidal concentration (MBC) assays demonstrated potent bactericidal activity, particularly against *E. coli* and *Klebsiella pneumoniae* (*K. pneumoniae*) at 0.3125 mg/mL. Furthermore, AgNPs synergistically enhanced the efficacy of conventional antibiotics in a species- and antibiotic-dependent manner. The strongest synergy was observed in *Enterococcus faecalis (E. faecalis)* (up to 9.84-fold with Colistin) and *Acinetobacter baumannii* (*A. baumannii*) (up to 5.11-fold with Ceftazidime), suggesting that AgNP-enhanced antibiotic efficacy varies depending on bacterial species, nanoparticle synthesis method, and antibiotic type.

**Conclusions:**

This study presents a novel and sustainable approach to tackling AMR by leveraging Sudanese yeast strains for the green synthesis of AgNPs. The findings underscore the potential of AgNPs as an effective antibacterial agent, both independently and in combination with conventional antibiotics, to combat MDR pathogens. By integrating microbiology and nanotechnology, this research offers a cost-effective and environmentally friendly solution for AMR mitigation. These findings provide a strong foundation for future clinical applications and public health interventions, particularly in resource-limited settings.

**Supplementary Information:**

The online version contains supplementary material available at 10.1186/s12866-025-04038-9.

## Introduction

Antimicrobial resistance (AMR) is a growing global public health crisis, responsible for an estimated 4.95 million deaths annually, with approximately 1.27 million deaths occurring in 2019 alone. Pathogens such as *Staphylococcus aureus* (*S. aureus*), *Escherichia coli (E. coli)*, and *P. aeruginosa* are major contributors to resistance [1]. The increasing prevalence of drug-resistant infections, particularly in low-income countries like Sudan, exacerbates the problem due to limited access to healthcare, inadequate infection control practices, and weak antibiotic regulation [2].

MDR bacterial infections are difficult to treat due to limited effective antibiotics. The misuse and overuse of antibiotics further fuel resistance, increasing the prevalence of these dangerous strains. In response, the World Health Organization (WHO) has emphasized the need for reducing antibiotic misuse and implementing integrated public health strategies to prevent bacterial resistance [3]. Given the urgency of the situation, alternative antimicrobial strategies are essential.

Although the exact mechanism of AgNPs’ antimicrobial action is not fully understood, several pathways have been proposed, including disruption of membrane potential and integrity through adhesion to the cell wall and membrane, activation of host immune responses, inhibition of biofilm formation, and generation of reactive oxygen species (ROS) [4]. AgNPs also induce lipid peroxidation, inhibit cytochromes in the electron transport chain, and interfere with cell wall synthesis. Additionally, they penetrate bacterial cells, damaging intracellular structures such as mitochondria, vacuoles, and ribosomes, while also disrupting biomolecules like proteins, lipids, and DNA [5].

A promising application of nanotechnology, AgNPs exhibit strong antimicrobial activity against both Gram-positive and Gram-negative multidrug-resistant (MDR) pathogens by destabilizing bacterial membranes, inducing oxidative stress, and interfering with vital cellular processes [6]. They further target bacterial efflux pumps and biofilm formation—key resistance mechanisms in *S. aureus* and *P.aeruginosa*—highlighting their potential as a powerful tool against antimicrobial resistance [7].

AgNPs can be synthesized through physical, chemical, and biological methods, each with distinct advantages and limitations. Physical methods are straightforward but often yield inconsistent nanoparticle sizes and are costly. Chemical synthesis produces smaller particles but involves harsh reaction conditions and environmental waste. In contrast, biological synthesis—using bacteria, fungi, yeast, algae, or plant extracts—offers a sustainable, biocompatible alternative with enhanced antibacterial properties. This eco-friendly approach has gained attention for its cost-effectiveness and scalability, making it a promising method for large-scale AgNP production. Notably, fungi-based AgNPs have shown minimal environmental toxicity, making them an attractive option [8].

Several fungal species, including *Fusarium*, *Aspergillus*, *Trichoderma*, *Verticillium*, *Rhizopus*, and *Penicillium*, have been extensively studied for AgNP biosynthesis, but research on single-celled yeasts remains limited [9]. Yeasts such as *Saccharomyces boulardii*, *Saccharomyces cerevisiae*, *Candida albicans*,* Candida utilis*, and *Candida lusitaniae* are of particular interest due to their ability to bioaccumulate metals and facilitate AgNP production via natural reduction and stabilization of silver ions [10]. Biogenic AgNPs have demonstrated potent antimicrobial activity against MDR strains of *S. aureus*, *P. aeruginosa*, *E. coli*, and *Acinetobacter*, proving effective even at low concentrations with minimal toxicity and negligible environmental impact [11, 12].

Despite biosynthesized AgNPs offer a sustainable alternative to chemical synthesis, their long-term environmental impact must be considered. AgNPs can accumulate in soil and water ecosystems, posing toxicity risks to aquatic organisms and microbial communities, thereby disrupting ecological balance. Additionally, prolonged human exposure may lead to cytotoxic effects, highlighting the need for safer and more sustainable synthesis methods [13].

As the global AMR crisis intensifies, it is critical to explore nanotechnology as part of an integrated public health approach. While AgNPs represent a powerful tool in combating AMR, they must be used alongside antimicrobial stewardship programs, infection control measures, and continuous surveillance to prevent further resistance [14]. A holistic strategy combining nanotechnology with established public health interventions is essential for effectively addressing AMR’s multifaceted challenges.

While fungal-mediated AgNP synthesis is well-documented, yeast-based synthesis remains underexplored. This study investigates *C. parapsilosis* for AgNP biosynthesis, leveraging its high metal bioaccumulation capacity and natural reduction mechanisms. Given its scalability, biocompatibility, and low toxicity, *C. parapsilosis* offers a promising eco-friendly approach for sustainable AgNP production, potentially contributing to AMR mitigation efforts.

## Materials and methods

### Isolation and identification of Fungi

In 2021, soil samples were collected from diverse plant zones in Khartoum State, Sudan, specifically from Halfia (north, dominated by guava trees) and Soba (south, bordered by nut and bean trees). Sampling was conducted at four different depths (0–5 cm, 5–10 cm, 10–15 cm, and 15–20 cm), covering both surface and subsurface layers [15]. To ensure sterility, uninoculated PDA and SDA plates (HiMedia, India) were included as negative controls in each batch. For fungal isolation, 10 g of soil was suspended in 90 mL of sterile 0.9% NaCl solution and homogenized using a magnetic stirrer (Bio Rad, USA) for 20–30 min. Serial tenfold dilutions (10⁻¹ to 10⁻⁵) were prepared, and 0.1 mL from each dilution was spread-plated onto PDA plates and incubated at 25 °C for 48–72 h under aerobic conditions. Pure fungal isolates were subsequently subcultured onto SDA plates (HiMedia, India) and incubated at 27 °C for 1–5 days to promote sporulation and maintain viability [16].

### Molecular identification using 18 S rRNA gene

DNA extraction was carried out using the CTAB method. Yeast material was suspended in a lysis buffer containing 200 mmol/L Tris-HCl (pH 8.0), 0.5% SDS, 250 mmol/L NaCl, and 25 mmol/L EDTA and homogenized using a Tissue Lyser LT (Qiagen, Germany) at 30 Hz for 2 min. The suspension was incubated at 100 °C for 15 min, followed by the addition of 150 µL of 3.0 mol/L sodium acetate (Sigma-Aldrich, USA) and cooling at -20 °C for 10 min. After centrifugation at 10,000 × g for 5 min using an Eppendorf 5424R Centrifuge (Eppendorf, Germany), the supernatant was sequentially extracted twice with phenol-chloroform-isoamyl alcohol (HiMedia, India), followed by a single extraction with chloroform. DNA was precipitated with isopropanol at -20 °C, washed with 70% ethanol, air-dried, and dissolved in 50 µL of ultrapure water.

The internal transcribed spacer (ITS) region was amplified using ITS1 (5′-CCGTAGGTGAACCTGCGG-3′) and ITS4 (5′-TCCTCCGCTTATTGATATGC-3′) primers (Sigma-Aldrich (Merck), USA). PCR amplification was performed in a 25 µL reaction volume containing:

1X PCR buffer, 200 µM of each dNTP,1.5 mM MgCl₂, 0.4 µM of each primer, 1.25 U of Platinum Taq High-Fidelity DNA Polymerase (Bio-Rad. USA), 5 µL of extracted DNA as a template.

PCR was carried out using a PCR machine (Bio-Rad, USA) under the following conditions:

Initial denaturation: 94 °C for 2 min.

35 Cycles of:

Denaturation: 94 °C for 30 s.

Annealing: 56 °C for 10 s.

Extension: 72 °C for 30 s.

Final extension: 72 °C for 2 min [16].

### Sequencing and analysis of the amplified DNA

PCR products were analyzed by electrophoresis on a 2% (w/v) agarose gel prepared in 1X TAE buffer (40 mM Tris-acetate, 1 mM EDTA, pH 8.0) and stained with ethidium bromide (0.5 µg/mL). Electrophoresis was conducted at 100 V for 45 min to separate DNA fragments. Each sample contained 5 µL of PCR product mixed with 1 µL of 6X gel loading dye (Bio-Rad, USA). A 1 kb Plus DNA Ladder (Bio-Rad, USA) was used to determine fragment sizes. Saccharomyces cerevisiae (RPMCC 9763) was included as a positive control.

Amplified DNA bands were excised from the gel and purified using the QIAquick Gel Extraction Kit (Qiagen, Germany), following the manufacturer’s silica column-based protocol. DNA concentration and purity were assessed using a NanoDrop 2000 spectrophotometer (Thermo Fisher Scientific, USA) at 260/280 nm. Sequencing was performed via Sanger sequencing using the BigDye™ Terminator v3.1 Cycle Sequencing Kit (Applied Biosystems, USA) on an ABI 3500 Genetic Analyzer (Applied Biosystems, USA). The ITS1 and ITS4 primers were used for bidirectional sequencing. Raw sequences were trimmed and assembled using BioEdit v7.2.5. Sequence similarity searches were performed using BLASTn against the NCBI GenBank nucleotide database to identify fungal species. Phylogenetic relationships were inferred using the Neighbor-Joining method in MEGA v11.0, with 1,000 bootstrap replicates to assess branch support [17].

### Biosynthesis of silver nanoparticles (AgNPs)

The *C. parapsilosis* isolates were evaluated for their ability to biosynthesize silver nanoparticles (AgNPs). A negative control was included by incubating sterilized cell-free filtrate without AgNO₃ under identical conditions to confirm that any observed color change or AgNP formation was due to silver reduction rather than other extracellular components.

The fungal biomass was prepared by culturing *C. parapsilosis* aerobically in Potato Dextrose Broth (PDB, HiMedia, India), formulated using 250 g of potato and 20 g of dextrose per liter of distilled water. The inoculum was standardized to an optical density (OD600) of 0.1 (equivalent to ~ 1 × 10⁶ CFU/mL) before inoculation into PDB. Cultures were incubated on an orbital shaker (Bio-Rad, USA) at 25 ± 2 °C with agitation at 120 rpm for 96 h.

Following incubation, the fungal biomass was harvested through vacuum filtration using Whatman No. 1 filter paper (GE Healthcare, Buckinghamshire, UK) and thoroughly washed three times with sterile distilled water to remove any residual medium components. A 10 g portion of the washed fungal biomass was suspended in 100 mL of sterilized double-distilled water and incubated for 48 h at 25 ± 2 °C in a 250 mL Erlenmeyer flask with continuous agitation at 120 rpm.

The resulting cell-free filtrate was obtained by filtration through Whatman No. 1 filter paper. This filtrate was then treated with a 1 mM solution of silver nitrate (AgNO₃, ≥ 99.9% purity, Sigma-Aldrich, USA) in a 250 mL Erlenmeyer flask and incubated at room temperature (~ 25 °C) in a dark environment to facilitate AgNP synthesis [16].

### Characterization of silver nanoparticles (AgNPs)

The absorption peaks of AgNP colloidal solutions were analyzed using multiple characterization techniques. Visual inspection revealed a gradual color change of the cell-free fungal filtrate from pale yellow to brown upon incubation with AgNO₃ solution under dark conditions. This color shift is attributed to Surface Plasmon Resonance (SPR), confirming the formation of silver nanoparticles.

To confirm AgNP synthesis, UV-Vis spectroscopy was conducted using a CARY-100 BIO UV-Vis Spectrophotometer (UV-Vis Systronic 2201) with a spectral resolution of 1 nm. Absorbance spectra were recorded in the range of 300–700 nm using a quartz cuvette (1 cm path length). The baseline was corrected using double-distilled water as a reference. To ensure that the observed SPR peak was specific to AgNP formation and not due to background absorption, two control spectra were recorded: fungal filtrate alone (without AgNO₃), to detect any intrinsic absorbance of biomolecules, and AgNO₃ solution alone (without fungal filtrate), to rule out any non-biological AgNP formation. The AgNP colloidal solution exhibited a distinct SPR peak at 419 nm, while the control samples showed no significant absorbance in this region.

X-ray diffraction (XRD) analysis was performed using a Philips X-ray generator (PW 3710/31, Philips, Japan). Transmission Electron Microscopy (TEM) analysis was conducted using a JEOL JSM 100CX TEM instrument (Jeol, Tokyo, Japan) operated at 200 kV. A drop of AgNP suspension was placed on a carbon-coated copper grid (200-mesh), air-dried at room temperature, and imaged at various magnifications to assess nanoparticle morphology and size distribution [16].

### Bacterial isolation from clinical samples

A total of 100 bacterial isolates were obtained from human clinical specimens collected from Bashair and Royal Care hospitals in Khartoum, Sudan, between July 8th, 2022, and September 2nd, 2022. The isolates included *K. pneumoniae*,* E. coli*,* A. baumannii*,* Pseudomonas spp.*,* S. Typhi*,* Serratia spp.*,* S. aureus*,* E. faecalis*,* Listeria monocytogenes*, and *Bacillus spp.*. Clinical specimens consisted of 36 urine samples, 22 wound swabs, and 42 blood cultures (Table S1).

### Re-identification of multidrug-resistant isolates

The bacterial isolates were cultured on Mueller-Hinton Agar, MacConkey Agar, and Blood Agar (all from HiMedia, India) and incubated at 37 °C for 18–24 h under aerobic conditions. Colonies were selected based on size, shape, pigmentation, lactose fermentation ability (MacConkey Agar), and hemolysis patterns (Blood Agar). Further identification was performed using Gram staining and biochemical tests, including catalase, oxidase, and coagulase assays (all from HiMedia, India). For precise species-level identification, the API 20E (bioMérieux, France) and API Staph (bioMérieux, France) test kits were used following the manufacturer’s instructions.

#### Macroscopic and microscopic identification

The isolates were examined for colony size, shape, color, and hemolytic properties on Blood Agar. Gram staining was performed using a Gram Stain Kit (HiMedia, India) to determine bacterial cell shape, arrangement, and Gram reaction [18].

#### Determination of multidrug resistance

Antimicrobial susceptibility testing was performed using the modified Kirby-Bauer disk diffusion method on Mueller-Hinton Agar (MHA, HiMedia, India), following the Clinical and Laboratory Standards Institute (CLSI) guidelines (2019). Bacteria were classified as multidrug-resistant (MDR) if they exhibited resistance to three or more antibiotic classes.

The tested antibiotics (all from HiMedia, India) included:

β-lactams: Amikacin (30 µg), Ampicillin (10 µg), Piperacillin (100 µg), Amoxicillin/Clavulanate (20/10 µg), Cefepime (30 µg), Ceftazidime (30 µg).

Fluoroquinolones: Ciprofloxacin (5 µg), Levofloxacin (5 µg).

Aminoglycosides: Gentamicin (10 µg).

Macrolides: Azithromycin (15 µg).

Sulfonamides: Trimethoprim/Sulfamethoxazole (25 µg).

Carbapenems: Imipenem (10 µg).

Zone diameters were measured and interpreted according to CLSI breakpoints [19].

#### Biochemical and API-based confirmation

Biochemical identification was performed using standard tests, including catalase, oxidase, coagulase, and sugar fermentation assays (all reagents from HiMedia, India). For precise species identification, API identification systems (bioMérieux, France) were used according to the manufacturer’s instructions:

API Staph System for the identification of Staphylococcus species.

API 20E Kit for the identification of Enterobacteriaceae and *P. aeruginosa*.

### Antimicrobial susceptibility test

#### Inoculum preparation

The McFarland Standard was prepared by mixing 0.5 mL of 1.175% barium chloride (BaCl₂) solution with 99.5 mL of 1% sulfuric acid (H₂SO₄) to achieve a turbidity standard equivalent to 0.5 McFarland. This standard serves as a reference to estimate bacterial concentrations in liquid suspensions. The absorbance was measured at 625 nm using a UV-Vis spectrophotometer (UV-Vis Systronic 2201), with an acceptable range of 0.08–0.13, corresponding to approximately 1.5 × 10⁸ CFU/mL.

#### Modified Kirby-Bauer method

The antibiotic susceptibility profile of the isolates was evaluated using the modified Kirby-Bauer disk diffusion method on Mueller-Hinton Agar (HiMedia, India), following the Clinical and Laboratory Standards Institute (CLSI) guidelines (2019). Standard antibiotic discs (HiMedia, India) were used as positive controls, while sterile distilled water served as a negative control.

MHA plates were inoculated with each bacterial isolate using sterile cotton swabs, ensuring even distribution. Antibiotic discs were carefully placed onto the prepared plates using sterile forceps to maintain sterility. The tested antibiotics included:

β-lactams: Amikacin (30 µg), Ampicillin (10 µg), Piperacillin (100 µg), Amoxicillin/Clavulanate (20/10 µg), Cefepime (30 µg), Ceftazidime (30 µg), Cephalexin (30 µg).

Fluoroquinolones: Ciprofloxacin (5 µg), Levofloxacin (5 µg).

Aminoglycosides: Gentamicin (10 µg).

Macrolides: Azithromycin (15 µg).

Sulfonamides: Trimethoprim/Sulfamethoxazole (25 µg).

Carbapenems: Imipenem (10 µg), Aztreonam (30 µg).

Other antibiotics: Vancomycin (30 µg), Tetracycline (30 µg), Clindamycin (2 µg), Colistin (10 µg), Chloramphenicol (30 µg), Nitrofurantoin (300 U).

The prepared plates were incubated at 37 °C for 18–24 h. After incubation, the diameter of the inhibition zones was measured in millimeters (mm) and compared with the CLSI standard breakpoint chart to classify bacterial isolates as resistant, intermediate, or susceptible [9].

### Antimicrobial activity of AgNPs

The antimicrobial activity of the synthesized silver nanoparticles (AgNPs) was tested against *E.coli* (ATCC 43890), *Enterococcus faecalis (E. faecalis)* (MTCC 2729), *S. aureus* (MTCC 96), *P. aeruginosa* (ATCC 27853), and previously isolated clinical bacterial strains.

Reference antibiotic discs of ciprofloxacin (5 µg), vancomycin (30 µg), and imipenem (10 µg) (all from Oxoid, HiMedia, India) were used as positive controls, while water extract without mycelia served as a negative control.

The antimicrobial activity was evaluated using the agar well diffusion method. Wells (9 mm in diameter) were created in Mueller-Hinton Agar (MHA, HiMedia, India) plates, and 100 µL, 500 µL, and 1000 µL of AgNP solutions were added to test low, medium, and high concentrations, respectively. A water extract without mycelia was used as a negative control to rule out any antimicrobial effects of fungal metabolites alone.

The plates were incubated at 37 °C for 48–72 h, after which inhibition zone diameters (in mm) were measured and compared against the positive control antibiotics [16].

### Minium inhibitory concentration (MIC) and minimum bactericidal concentration (MBC) determination

The Minimum Inhibitory Concentration (MIC) and Minimum Bactericidal Concentration (MBC) of AgNPs were determined using the broth dilution method, following CLSI M07-A8 guidelines.

Serial dilutions of AgNPs were prepared in Brain Heart Infusion (BHI) broth (HiMedia, India), ranging from 5 mg/mL to 0.156 mg/mL. The bacterial suspension was standardized to 10⁸ CFU/mL using a 0.5 McFarland standard, and 100 µL of each dilution was inoculated into sterile 96-well microplates containing BHI broth. A non-inoculated broth served as a negative control.

The microplates were incubated at 37 °C for 24 h, and the MIC was defined as the lowest AgNP concentration with no visible bacterial growth.

For MBC determination, 50 µL from wells showing no visible growth was spread onto Mueller-Hinton Agar (HiMedia, India) and incubated at 37 °C for 24 h. The MBC was recorded as the lowest AgNP concentration that resulted in a 99.9% bacterial reduction [19].

### Synergistic effects of AgNPs with resistant antibiotics

The synergistic effects of AgNPs with commonly used antibiotics were evaluated using the disk diffusion method. A 20 µL solution of AgNPs was applied to standard antibiotic disks, which were placed on MHA plates inoculated with test organisms. The plates were incubated at 25 °C for 24–48 h, and the diameters of the inhibition zones were measured. The experiment was repeated three times for consistency [16]. To quantify the enhancement, we calculated an area-based fold increase in the inhibitory zones for each replicate using the equation:

$$\:\text{A}\:={\left(\frac{\text{Y}}{\text{X}}\right)}^{2}-\:1\:$$

Where A = Area of fold Increase, Y = Inhibition zone of Combination between antibiotic and AgNP3, X = Inhibition zone of antibiotic alone. In this equation, the squared ratio accounts for the two-dimensional nature of the inhibition zones, while the subtraction of 1 serves to normalize the value so that a fold increase of 0 indicates no change compared to the control. Positive values reflect a synergistic enhancement of the antibiotic effect due to the AgNP coating [20].

### Membrane integrity and permeability in vivo activity

The integrity of the bacterial cell membrane was evaluated by measuring the release of nucleic acids (DNA and RNA), which absorb at 260 nm, following exposure to specific concentrations of AgNPs. The bacterial suspension was treated with AgNPs, and samples were collected at defined time intervals. Absorbance measurements at 260 nm were recorded using a UV-Vis spectrophotometer (UV-Vis Systronic 2201) to assess membrane leakage. To further assess membrane permeability changes, O-nitrophenyl β-D-galactopyranoside (ONPG) (Sigma-Aldrich, USA) was used as a β-galactosidase substrate. The hydrolysis of ONPG was quantified by measuring absorbance at OD420 over time, indicating membrane disruption [21].

### Statistical analysis

All analyses were performed using R 4.3.3 [22], with visualization via ggplot2 [23]. Normality was assessed using the Shapiro-Wilk test. Categorical variables were expressed as percentages, while quantitative data were presented as mean ± standard deviation or median. Group comparisons were conducted using the Kruskal-Wallis test for continuous variables, the chi-square test for categorical variables, and Fisher’s exact test when expected frequencies were low. Dunn’s post hoc test with Bonferroni correction was used for pairwise comparisons, and the Wilcoxon test assessed whether median fold increases differed from zero. Hierarchical clustering with Euclidean distance and complete linkage was used to analyze antibiotic resistance patterns, visualized via heatmaps package in R. A *p*-value of ≤ 0.05 was considered statistically significant.

## Result

### Fungi isolation and identification

**Fig. 1:**
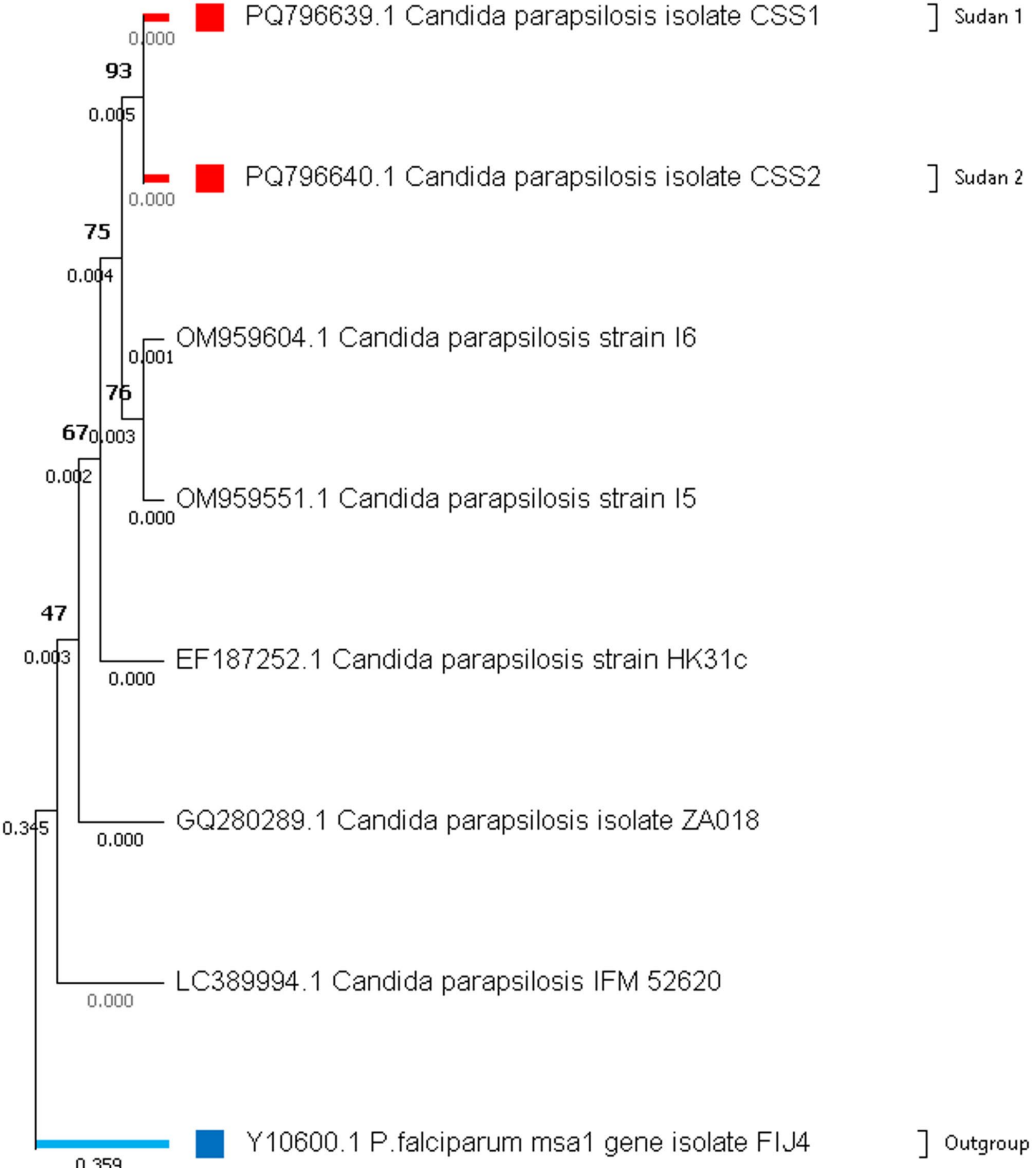
Phylogenetic tree of *C. parapsilosis* isolates based on the 18 S rRNA gene. The tree was constructed using the Neighbor-Joining method in MEGA 11. The newly identified isolates from Sudan (CSS1 and CSS2) are highlighted in red, while the outgroup (*Plasmodium falciparum* msa1 gene isolate FIJ4) is shown in blue. Bootstrap values are displayed at the nodes. The scale bar represents genetic distance

Two strains of *C. parapsilosis*, designated CSS1 (accession number PQ796639) and CSS2 (accession number PQ796640), were isolated from soil samples collected in Khartoum, Sudan. A phylogenetic tree was constructed to confirm their taxonomic identification (Fig. 1). These fungal isolates were subsequently utilized for the biosynthesis of silver nanoparticles (AgNPs) using silver nitrate (AgNO₃) as the precursor, and their antibacterial activity was evaluated. *Plasmodium falciparum* msa1 gene, isolate FIJ4, partial.

### Biosynthesis of AgNPs

The synthesis of AgNPs resulted in distinct solution colors corresponding to three different concentrations: low concentration (L), medium concentration (M), and high concentration (H) (S Fig. 1).

### Characterization of AgNPs

**Fig. 2:**
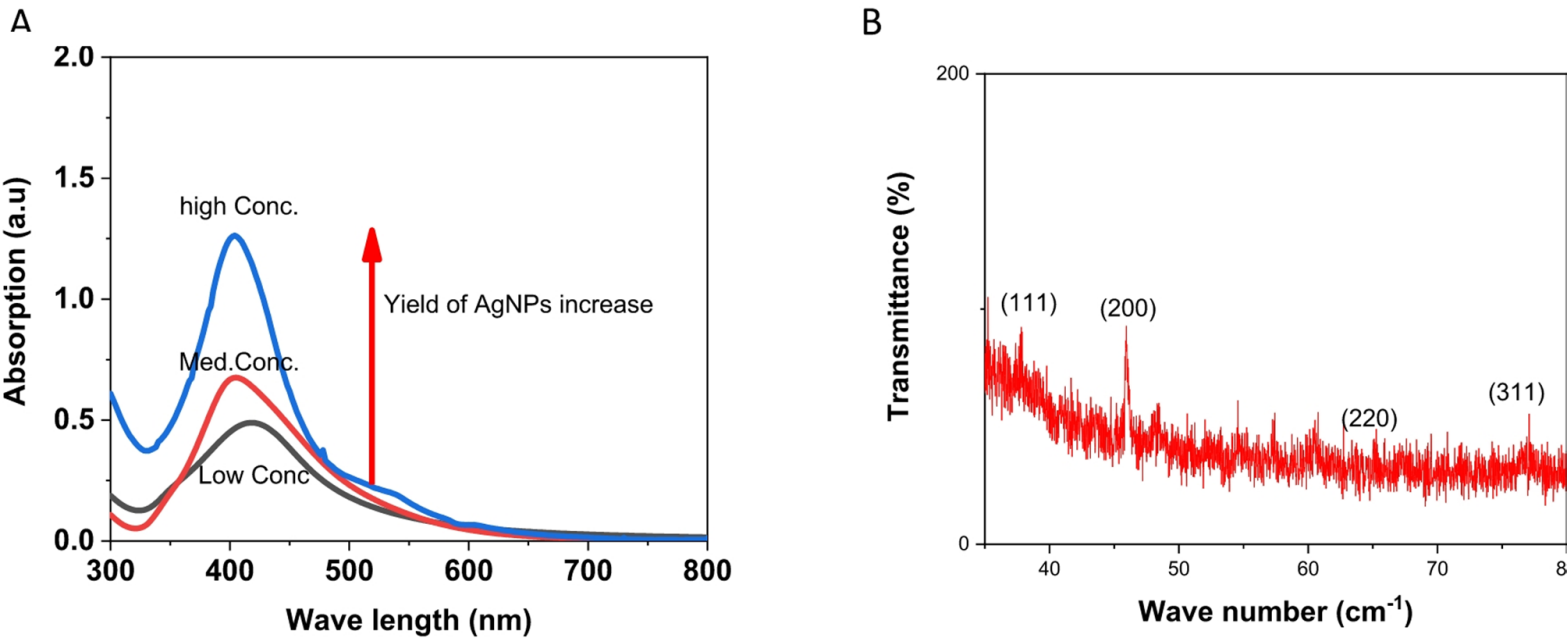
**A**: UV-visible absorption spectra of biosynthesized AgNPs at varying concentrations following exposure to silver nitrate solution. **B**: XRD diffraction pattern of biosynthesized silver nanoparticles (AgNPs)

The analysis of the synthesized silver nanoparticles (AgNPs) using various methods revealed distinct findings. As shown in (Fig. 2A), the UV-Vis absorption spectra of AgNPs synthesized under different conditions exhibit surface plasmon resonance (SPR) peaks within the range of 400–450 nm, confirming the formation of AgNPs. Variations in peak intensity, position, and width indicate differences in nanoparticle size, uniformity, and aggregation. A sharper, more intense peak (blue curve) suggests the presence of smaller, more uniform nanoparticles, while broader or less intense peaks (red and black curves) indicate larger particles or increased aggregation. These results highlight how synthesis conditions affect the optical and structural properties of the AgNPs.

The X-ray diffraction (XRD) analysis, presented in (Fig. 2B), showed distinct diffraction peaks at 2θ angles of 37.8°, 45.9°, 65.1°, and 77.01°, corresponding to the crystallographic planes (111), (200), (220), and (311), confirming the crystalline nature of the AgNPs.

**Fig. 3:**
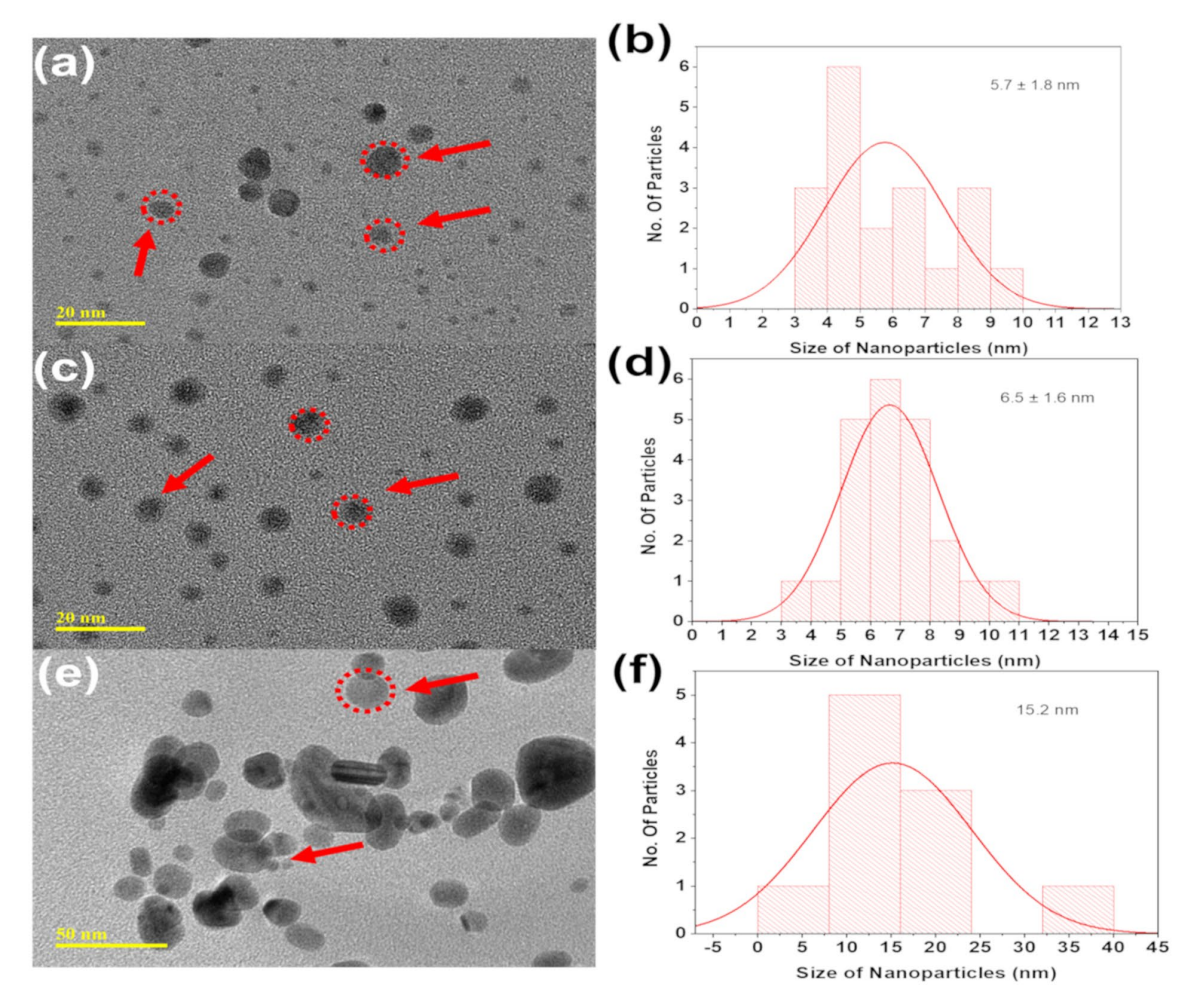
TEM images and corresponding histograms of biosynthesized AgNPs: (**a**) AgNPs at low concentration with (**b**) their particle size distribution histogram, (**c**) AgNPs at medium concentration with (**d**) their histogram, and (**e**) AgNPs at high concentration with (**f**) their histogram

The TEM images and histograms in (Fig. 3) illustrate the impact of precursor material concentration on the characteristics of silver nanoparticles (AgNPs). At low concentrations, the nanoparticles appear smaller with less aggregation, while the histogram indicates moderate size uniformity (Fig. 3a, b).

**Fig. 4:**
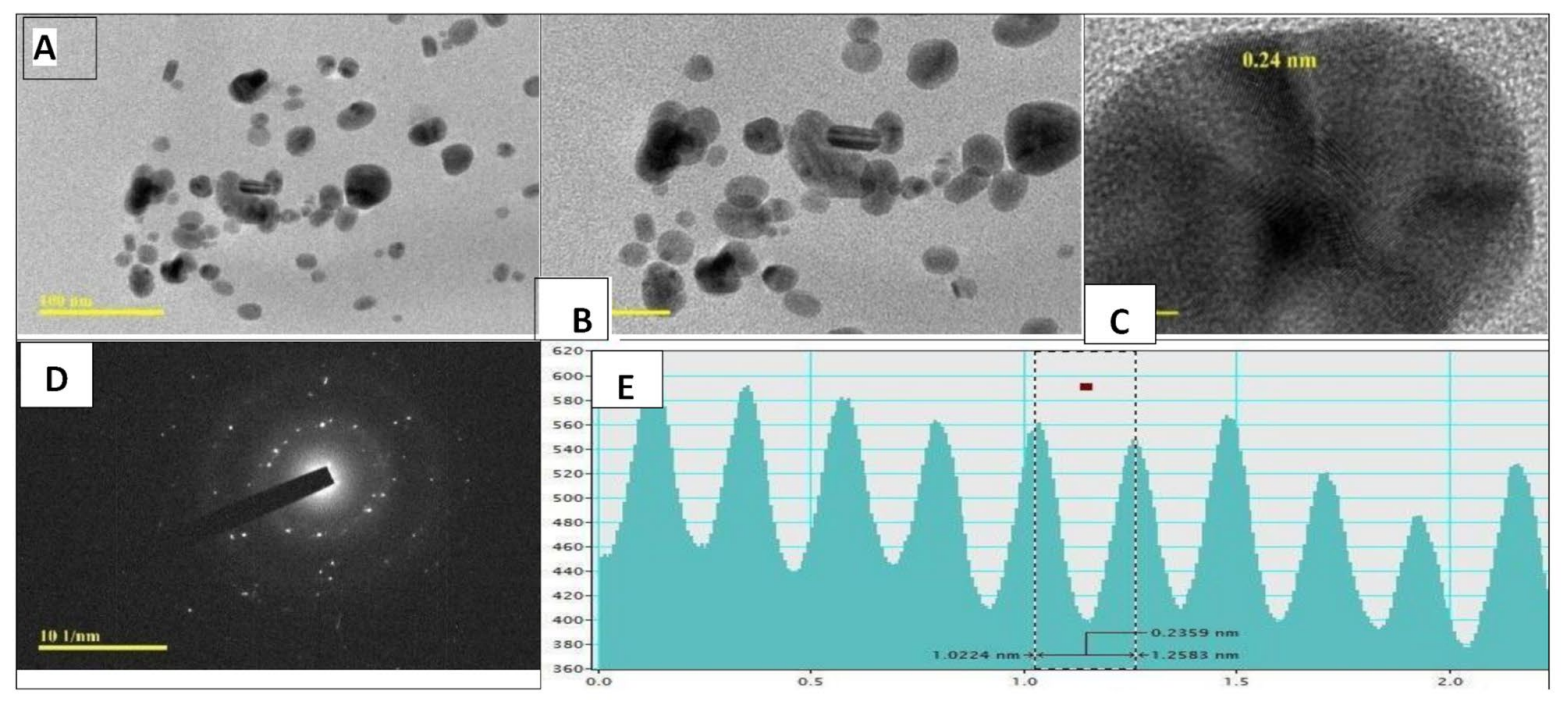
HRTEM analysis of biosynthesized AgNPs: (**A**) HRTEM image with a scale bar of 100 nm, (**B**) magnified image with a 50 nm scale bar, (**C**) high-resolution image with a 5 nm scale bar, (**D**) selected area electron diffraction (SAED) pattern, and (**E**) line profile showing interplanar lattice fringes

Medium concentrations result in more uniform and well-dispersed nanoparticles with minimal aggregation, as reflected by the narrower size distribution in the histogram (Fig. 3c, d). At high concentrations, significant aggregation is evident, leading to larger, irregular clusters, with the histogram showing a wider size distribution and reduced control over particle size (Fig. 3e, f). These findings highlight that medium precursor concentrations are optimal for achieving a balance between particle size uniformity and minimal aggregation. High concentrations, on the other hand, lead to excessive aggregation, which may negatively affect the functional performance of the nanoparticles in applications such as catalysis, antimicrobial activity, or optical technologies. The results emphasize the importance of carefully controlling precursor concentrations during synthesis, particularly when scaling up production, to ensure consistent nanoparticle characteristics.

(Fig. 4) presents High-Resolution Transmission Electron Microscopy (HRTEM) analysis, confirming the successful biosynthesis of AgNPs with the following key findings: Morphology (100 nm scale): The AgNPs are predominantly spherical or nearly spherical, with some aggregation, and their sizes are within the nanometer range (Fig. 4A). Higher Magnification (50 nm scale): At higher magnification, the spherical shape of the nanoparticles is confirmed, offering a detailed visualization of individual particles (Fig. 4B). Crystalline Structure (5 nm scale): Lattice fringes observed at high magnification reveal the crystalline nature of the AgNPs, with a lattice spacing of 0.24 nm corresponding to the (111) plane of metallic silver (Fig. 4C).

Selected Area Electron Diffraction (SAED) Pattern: The bright concentric rings in the diffraction pattern indicate a polycrystalline structure, confirming the metallic silver composition of the nanoparticles (Fig. 4D). Line Profile Analysis: Numerical analysis of lattice fringes shows interplanar distances (e.g., 0.2359 nm and 0.2588 nm), matching theoretical values for silver’s crystal planes (Fig. 4E). These findings validate that the synthesized AgNPs are highly crystalline and hold significant potential for various applications.

### Antibiotic susceptibility of isolated Bacteria

The antibiotic susceptibility analysis identified multidrug-resistant (MDR) bacterial strains for potential use in evaluating biosynthesized nanoparticles (Table S2 &Table S3). Ten pathogenic bacteria were tested against 20 antibiotics, revealing widespread resistance (Approximative Kruskal-Wallis Test, *p* < 0.0001). The resistance cutoff was set to ≥ 40 where bacteria (Table S4), and all bacterial species met the MDR criteria (Table S5), with *E. coli*, *K. pneumoniae*, *P. aeruginosa*, and *A. baumannii* exhibiting resistance to more than 50% of antibiotics. Even *Bacillus cereus* and *Listeria monocytogenes* (*L. monocytogenes*), which displayed lower resistance rates, were resistant to at least 4–6 antibiotics in three classes.

**Fig. 5:**
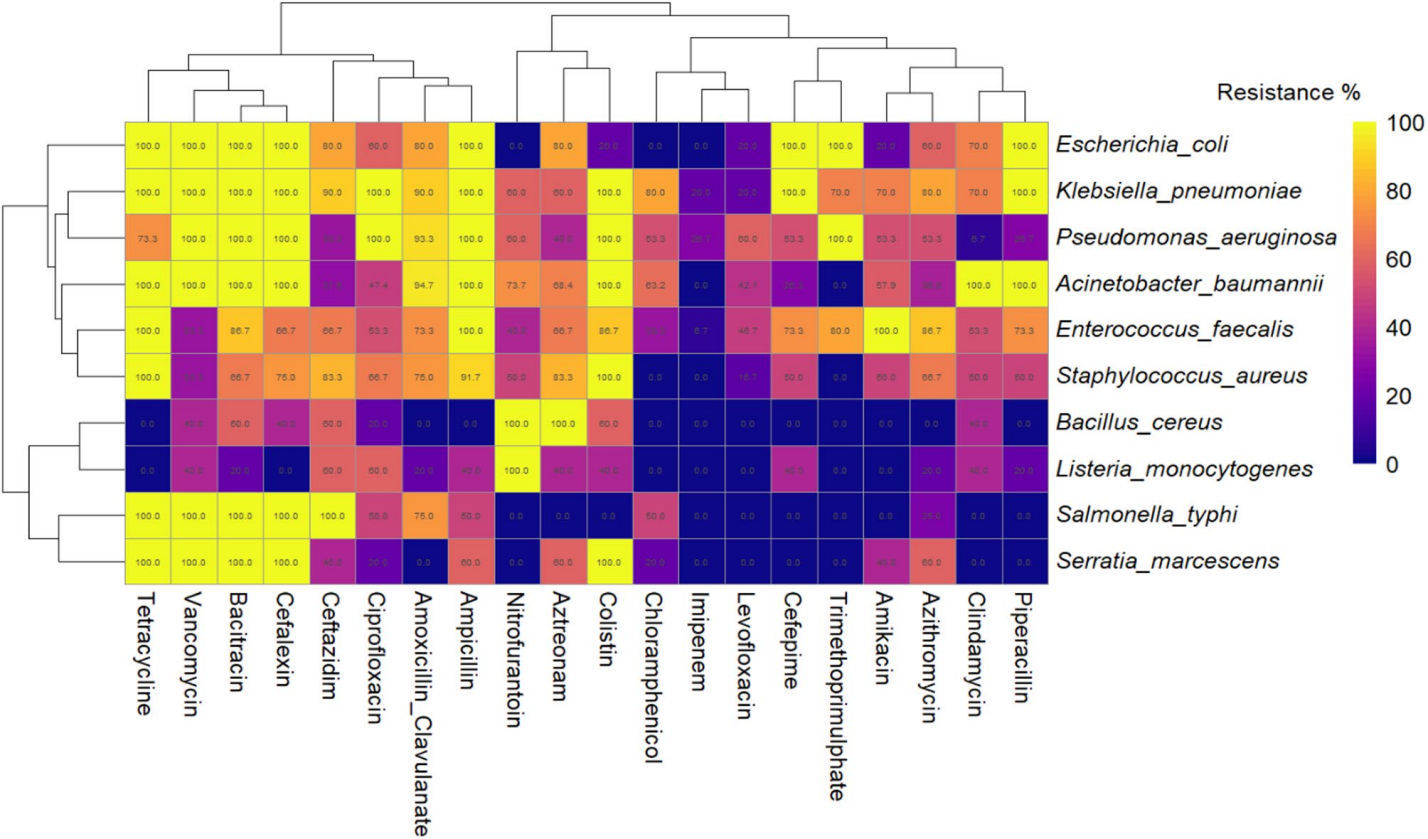
Clustering Analysis of Antibiotic Resistance Patterns in Multidrug-Resistant Bacterial Pathogens

Clustering analysis (Fig. 5) highlighted high resistance levels, particularly to β-lactam antibiotics. *K. pneumoniae* and *P. aeruginosa* showed > 90% resistance to third-generation cephalosporins, while *A. baumannii* displayed 100% resistance to Vancomycin, Piperacillin, and Clindamycin. Conversely, *L. monocytogenes* had lower resistance rates with susceptibility to most tested antibiotics except Azithromycin (20%) and Piperacillin (20%).

*E. coli*,* K. pneumoniae*, and *P. aeruginosa* exhibited extensive resistance (> 80%) to commonly prescribed antibiotics, including Ampicillin, Amoxicillin-Clavulanate, and Ciprofloxacin, underscoring their role in antimicrobial resistance (AMR) dissemination. *A. baumannii* displayed complete resistance (100%) to Vancomycin, Piperacillin, and Clindamycin, further establishing its multidrug-resistant profile.

Despite prevalent resistance, some antibiotics remained effective. Imipenem exhibited < 20% resistance in most species, while Nitrofurantoin showed complete efficacy against *E. coli* and *S.typhi*. *Bacillus cereus* was broadly susceptible except for complete resistance to Aztreonam. These findings highlight MDR bacteria’s role in antimicrobial resistance in Sudan and justify their selection for testing silver nanoparticles (AgNPs) synthesized using *C. parapsilosis*.

### Antimicrobial activity of AgNPs

**Fig. 6:**
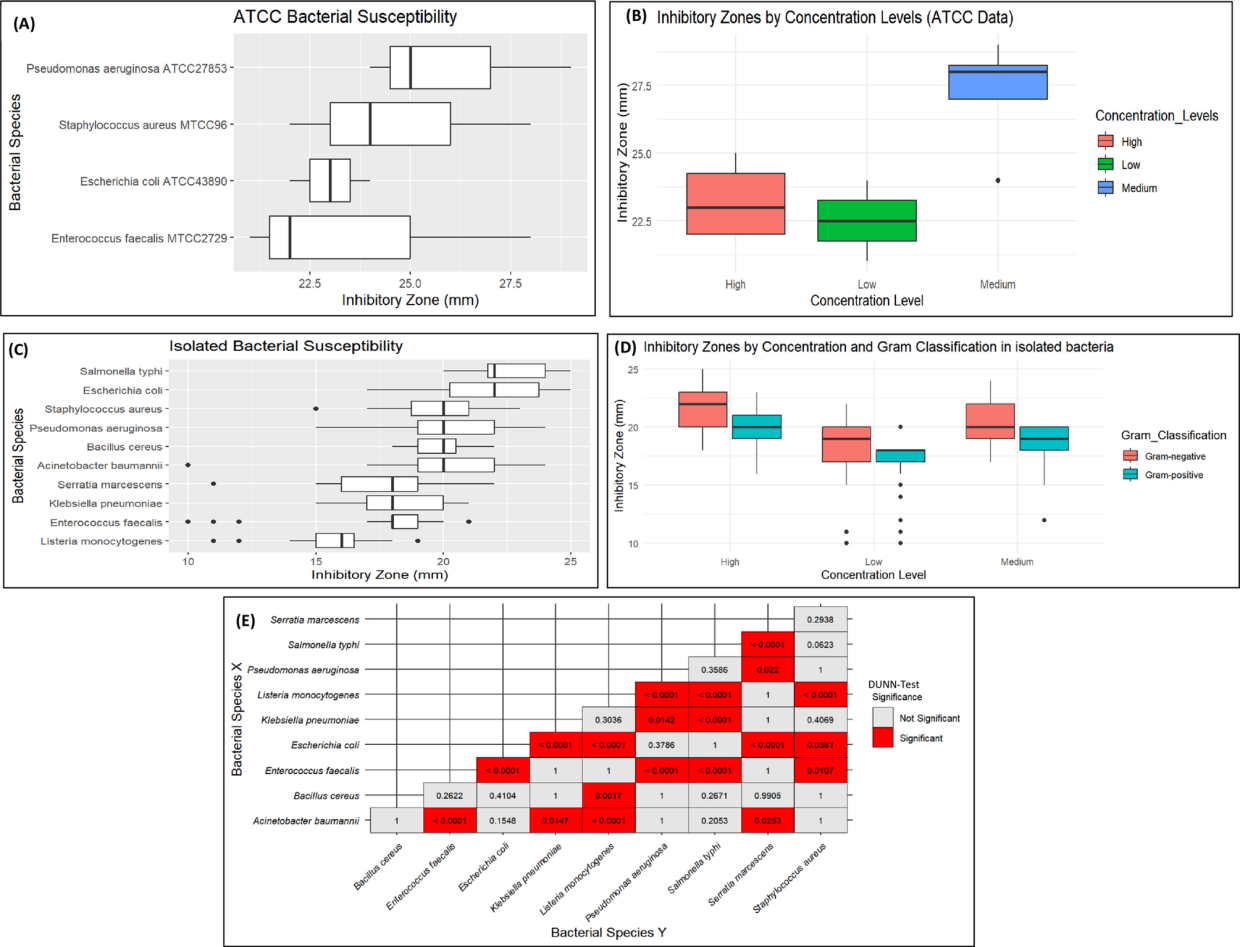
Antimicrobial susceptibility and inhibitory zone analysis of atcc and clinically isolated bacteria. (**A**) Susceptibility of ATCC Bacteria; (**B**) Effect of Concentration Levels on Inhibitory Zones in ATCC Reference Data; (**C**) Susceptibility of Clinically Isolated Bacteria; (**D**) Effect of Concentration Levels and Gram Classification on Inhibitory Zones in Clinically Isolated Bacteria; (**E**) Pairwise Comparison Matrix of Silver Nanoparticles (AgNPs) Antimicrobial Activity in Clinically Isolated Bacteria (significant differences in red)

Silver nanoparticles (AgNPs) exhibited antibacterial activity against both ATCC reference strains and clinically isolated bacteria, with inhibition zones varying across different concentrations. For ATCC strains.

(Fig S 2) (Fig. 6A-B) (Table 1), medium concentrations (250 mg/mL) demonstrated the highest efficacy, with inhibition zones averaging 27.3 ± 2.2 mm. The largest inhibition zone was observed for *P. aeruginosa* (ATCC 27853) at 29 mm, followed by *E. faecalis* (MTCC 2729) and *S. aureus* (MTCC 96) at 28 mm each. High concentrations (500 mg/mL) resulted in slightly lower inhibition zones (23.2 ± 1.5 mm on average), possibly due to nanoparticle aggregation affecting diffusion. The lowest inhibition was observed at 100 mg/mL (22.5 ± 1.3 mm). The Kruskal-Wallis test for ATCC strains revealed a statistically significant difference in inhibitory zones across concentration levels (*p* = 0.04516). However, post-hoc Dunn’s test with Bonferroni correction showed that none of the pairwise comparisons were statistically significant (*p* > 0.05). A trend was observed between low and medium concentrations (*p* = 0.0504), which may be due to the limited number of bacterial species in this group.

For clinically isolated bacteria (Fig. 6C), the highest inhibition zones were observed at high concentrations (21.2 ± 1.92 mm), with *S. typhi* (24.5 ± 0.58 mm) and *E. coli* (23.8 ± 0.79 mm) being the most susceptible (Fig S 3) (Table 2). Medium concentrations showed comparable efficacy (19.69 ± 1.92 mm), particularly against *P. aeruginosa* (20.6 ± 1.50 mm) and *A. baumannii* (20.05 ± 1.13 mm). Low concentrations exhibited the least antibacterial activity (17.62 ± 2.6 mm), with *S. marcescens* showing the lowest inhibition (13.4 ± 3.58 mm). The Kruskal-Wallis test for clinically isolated bacteria demonstrated a highly significant difference across concentration levels (*p* < 0.0001). Dunn’s post-hoc test confirmed significant differences between high vs. low (*p* < 0.0001), high vs. medium (*p* = 0.000012), and low vs. medium (*p* < 0.0001), indicating a concentration-dependent effect of AgNPs.

The antibacterial activity of AgNPs was further analyzed by comparing their effects on Gram-negative and Gram-positive clinically isolated bacteria at different concentration levels (Fig. 6D). For Gram-negative clinically isolated bacteria, the Kruskal-Wallis test revealed a highly significant difference across concentration levels (*p* < 0.0001). The highest inhibition was observed at high concentrations (21.9 ± 1.73 mm), followed by medium concentrations (20.3 ± 1.74 mm) and low concentrations (18.1 ± 2.59 mm). Post-hoc analysis showed significant differences between high vs. low (*p* < 0.0001), high vs. medium (*p* = 0.000224), and low vs. medium (*p* = 0.0000141), confirming a concentration-dependent inhibitory effect. Similarly, for Gram-positive clinically isolated bacteria, a significant concentration-dependent response was observed (*p* < 0.0001), where high concentrations showed the largest inhibition zones (20.1 ± 1.68 mm), followed by medium concentrations (18.6 ± 1.74 mm) and low concentrations (16.8 ± 2.47 mm). Post-hoc analysis indicated significant differences between high vs. low (*p* < 0.0001), high vs. medium (*p* = 0.011), and low vs. medium (*p* = 0.00145).

Furthermore, a pairwise comparison of bacterial susceptibility using Dunn’s test revealed statistically significant differences among various bacterial species (Fig. 6E). The heatmap illustrates the significance levels of inhibitory zone differences. These differences reinforce the variability in bacterial responses to AgNPs, emphasizing the need for species-specific considerations in antimicrobial applications.

**Table 1:** Inhibitory effect of biosynthesized AgNPs at high, medium, and low concentrations against ATCC reference bacterial strains

Bacterial Category	Bacterial Species	Inhibitory Zones Concentration		
		Low (mm)	Medium (mm)	High (mm)
ATCC	*E. faecalis MTCC2729*	21.00	28.00	22.00
	*E.coli ATCC43890*	23.00	24.00	22.00
	*P. aeruginosa ATCC27853*	24.00	29.00	25.00
	*S. aureus MTCC96*	22.00	28.00	24.00
Total Mean ± StD (ATCC reference)		22.5 ± 1.3	27.3 ± 2.2	23.2 ± 1.5

**Table 2:** Inhibitory effect of biosynthesized AgNPs at high, medium, and low concentrations against various Gram-Positive and Gram-Negative bacterial species

Bacterial Category	Bacterial Species	Inhibitory Zones Concentration		
		Low (mm)	Medium (mm)	High (mm)
Gram -ve Bacteria	*A. baumannii*	18.37 ± 2.34	20.05 ± 1.13	22 ± 1.15
	*E. coli*	19.5 ± 1.58	22.4 ± 0.70	23.8 ± 0.79
	*K. pneumoniae*	16.7 ± 1.34	18.5 ± 1.08	20.1 ± 0.74
	*P. aeruginosa*	18.53 ± 1.77	20.6 ± 1.50	21.67 ± 1.11
	*S. typhi*	20.75 ± 0.96	22.25 ± 0.50	24.5 ± 0.58
	Total Mean ± StD (Gram -ve)	18.1 ± 2.59	20.3 ± 1.74	21.9 ± 1.73
	*Serratia marcescens*	13.4 ± 3.58	18.4 ± 1.14	19.6 ± 1.52
Gram + ve Bacteria	*Bacillus cereus*	18.4 ± 0.89	20 ± 00	21 ± 0.71
	*E. faecalis*	16.13 ± 2.72	18.4 ± 0.74	19.4 ± 0.74
	*Listeria monocytogenes*	14.2 ± 1.92	15.2 ± 1.92	17.4 ± 1.34
	*S. aureus*	18.17 ± 1.40	19.75 ± 0.45	21.58 ± 1.00
	**Total Mean ± StD (Gram + ve)**	16.8 ± 2.47	18.6 ± 1.74	20.1 ± 1.68
Total Mean ± StD (Isolated Bacteria)		17.62 ± 2.6	19.69 ± 1.92	21.2 ± 1.92

### Antimicrobial activity of silver nanoparticles (AgNPs) via tube dilution method for MIC and MBC

Silver nanoparticles (AgNPs) exhibited potent bactericidal activity against all tested Gram-negative bacteria, as indicated by MBC/MIC ratios ≤ 1 (Table 3). *E. coli* and *K. pneumoniae* demonstrated the highest sensitivity, with both MIC and MBC at 0.3125 mg/mL (MBC/MIC = 1).

*P. aeruginosa* and *A. baumannii* also showed strong bactericidal effects (MBC/MIC = 0.5), with MIC values of 1.25 mg/mL and MBC values of 0.625 mg/mL. Similarly, *S. typhi* and *S. marcescens* displayed MBC/MIC ratios of 0.5, confirming their susceptibility to AgNPs.

AgNPs also exhibited variable antibacterial activity against Gram-positive bacteria (Table 4). *S. aureus* had an MBC/MIC ratio of 1, confirming bactericidal activity. The most susceptible Gram-positive bacteria were *L. monocytogenes* and *B. cereus* (MBC/MIC = 0.5), suggesting potent bactericidal effects. In contrast, *E. faecalis* had an MBC/MIC ratio of 2, suggesting that AgNPs may exert bacteriostatic effects at lower concentrations.

These findings highlight the strong antibacterial potential of AgNPs, particularly against highly susceptible Gram-negative bacteria such as *E. coli* and *K. pneumoniae*, as well as Gram-positive bacteria *B. cereus* and *L. monocytogenes*. However, the variable response among Gram-positive species suggests species-dependent differences in susceptibility to AgNPs.

**Table 3:** Minimum inhibitory concentration (MIC) and minimum bactericidal concentration (MBC) of AgNPs (mg/mL) against Gram-Negative bacteria

Bacterial Species	MIC (mg/mL)	MBC (mg/mL)	MBC/MIC Ratio
*E. coli*	0.3125	0.3125	1
*K. pneumoniae*	0.3125	0.3125	1
*P. aeruginosa*	1.25	0.625	0.5
*A.r baumannii*	1.25	0.625	0.5
*S. typhi*	0.625	0.3125	0.5
*Serratia marcescens*	0.625	0.3125	0.5

**Table 4:** Minimum inhibitory concentration (MIC) and minimum bactericidal concentration (MBC) of AgNPs (mg/mL) against Gram-Positive bacteria

Bacterial Species	MIC (mg/mL)	MBC (mg/mL)	MBC/MIC Ratio
*S. aureus*	0.625	0.625	1
*E. faecalis*	0.625	1.25	2
*Listeria monocytogenes*	0.625	0.3125	0.5
*Bacillus cereus*	0.3125	0.1562	0.5

### Synergistic activity of AgNPs coated on antibiotics against resistant bacteria

In this study, we evaluated the enhancement of antibacterial activity when conventional antibiotics were coated with silver nanoparticles (AgNPs) against a range of resistant bacterial strains. Overall, our analyses demonstrate that AgNP coating significantly boosts antibiotic efficacy in a manner that is dependent on both the bacterial species and the specific antibiotic used. Detailed results and statistical comparisons are provided in Tables S6, S7, and S8.

Our results reveal distinct patterns of synergy across bacterial species. For instance, in *L. monocytogenes*, AgNP-coated Ceftazidime and Colistin produced mean fold increases of 3.09 ± 0.20 and 2.85 ± 0.34, respectively (both *p* = 0.0477), indicating a moderate enhancement. In *S. aureus*, the synergistic effect was even more pronounced; Colistin and Ceftazidime resulted in mean fold increases of 4.57 ± 1.22 and 3.92 ± 1.56 (*p* ≈ 0.0024 for both), with additional significant increases observed for Tetracycline (2.82 ± 0.70, *p* = 0.00239) and Chloramphenicol (2.25 ± 0.57, *p* = 0.00238). In the case of *K. pneumoniae*, antibiotics such as Tetracycline (3.53 ± 2.18, *p* = 0.00557), Ampicillin (2.74 ± 0.72, *p* = 0.00573), and Erythromycin (2.53 ± 0.88, *p* = 0.00557) showed enhanced activity with AgNP coating, while in *E. coli*, Colistin achieved a fold increase of 2.91 ± 0.88 (*p* = 0.00548) and both Tetracycline and Nitrofurantoin yielded modest enhancements (1.90 ± 0.55 and 1.81 ± 0.45; *p* = 0.00586 each).

A particularly strong synergistic effect was observed in *E. faecalis*, where AgNP-coated Colistin resulted in a remarkable fold increase of 9.84 ± 4.53 (*p* = 0.00070). Other antibiotics in this species, including Ceftazidime (4.17 ± 2.21, *p* = 0.00067), Ampicillin (3.54 ± 2.08, *p* = 0.00070), Cefepime (3.25 ± 0.60, *p* = 0.00069), Tetracycline (2.61 ± 0.78, *p* = 0.00070), Chloramphenicol (2.05 ± 0.69, *p* = 0.00066), and Erythromycin (0.99 ± 0.47, *p* = 0.00070), all exhibited statistically significant enhancements. In *A. baumannii*, AgNP coating markedly increased the efficacy of Ceftazidime and Colistin (with fold increases of 5.11 ± 2.24, *p* = 0.00014 and 4.32 ± 0.94, *p* = 0.00043, respectively), and a similar trend was observed in *P. aeruginosa*, where significant improvements were recorded for several antibiotics, including Ceftazidime (4.11 ± 1.13, *p* = 0.00070) and Colistin (2.92 ± 0.89, *p* = 0.00071).

When differences across bacterial species were statistically compared using a Kruskal-Wallis test, we found significant overall variability in fold increases (H = 64.21, df = 9, *p* < 0.0001). Post hoc pairwise comparisons (via Dunn’s test with Bonferroni correction) indicated that *A. baumannii* exhibited significantly higher fold increases than *E. faecalis* (p-adj < 0.0001), *Listeria monocytogenes* (p-adj = 0.00001086), *K. pneumoniae* (p-adj = 0.0137), *S. typhi* (p-adj = 0.0396), and *S. aureus* (p-adj = 0.0223). Conversely, *E. faecalis* showed higher fold increases than *E. coli* (p-adj = 0.0276) and *P. aeruginosa* (p-adj < 0.0001), and *L. monocytogenes* had a greater fold increase than *P. aeruginosa* (p-adj = 0.0120).

In addition to these species-specific effects, we also observed significant variability in the fold increase when comparing different antibiotics. A Kruskal-Wallis test across antibiotics revealed a highly significant difference (H = 414.04, df = 9, *p* < 0.0001). Post hoc comparisons using Dunn’s test with Bonferroni correction (see Table S8) demonstrated that Ampicillin, for example, showed significantly higher fold increases compared to Ciprofloxacin (p-adj = 0.0373) and Nitrofurantoin (p-adj = 0.0152), and markedly superior performance relative to Cefepime, Ceftazidime, Colistin, and Tetracycline (all p-adj < 0.0001). Further, several pairwise comparisons—such as between Cefepime and Colistin (p-adj = 0.00017), and between Ceftazidime and Chloramphenicol or Ciprofloxacin (both p-adj < 0.0001)—underscore the significant variability in synergy depending on the antibiotic used.

In summary, our integrated analysis confirms that coating antibiotics with AgNPs significantly enhances their antibacterial activity, with the magnitude of this effect varying according to both the bacterial species and the antibiotic. The fold increase metric—derived by squaring the ratio of inhibition zones and subtracting 1 to set the control baseline—proved to be a robust indicator of synergistic enhancement. Notably, *A. baumannii* and *E. faecalis* displayed the most pronounced improvements, while significant differences were also evident among the antibiotics tested, particularly favoring Ampicillin. These findings highlight the potential of AgNP-based formulations as a promising strategy to potentiate conventional antibiotics against resistant pathogens.

### Confirmatory the antibacterial activity of AgNPs

**Fig. 7:**
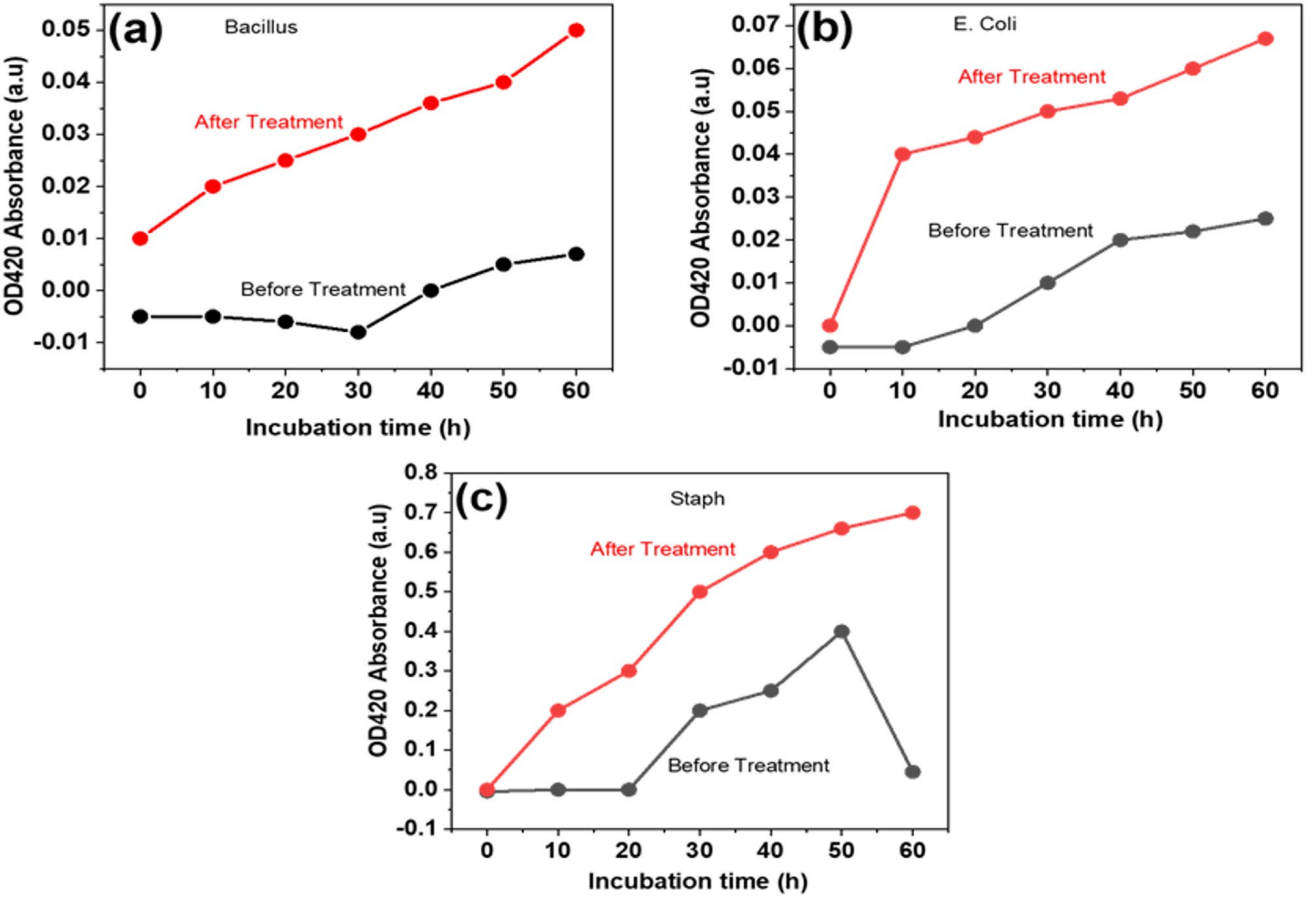
Line chart depicting the membrane permeability of representative bacterial isolates before and after treatment with green-synthesized AgNPs: (**a**) *Bacillus*, (**b**) *E. coli*, and (**c**) *S. aureus*

The antibacterial effect of AgNPs on bacterial membrane integrity was assessed using three line graphs comparing treated and untreated bacterial cells. The data showed significant alterations in membrane integrity following AgNP treatment. Across all graphs, the red lines (post-treatment) demonstrated a marked increase in the measured parameter, such as membrane permeability or damage, compared to the gray lines (control), indicating the disruptive effect of AgNPs on bacterial membranes. In the (Fig. 7a), membrane integrity steadily increased after AgNP treatment, suggesting a continuous disruption of the bacterial membranes.

In (Fig. 7b), a sharp rise in membrane damage was noted immediately following AgNP treatment, followed by a slower yet sustained increase, while the control group showed minimal changes. The (Fig. 7c) exhibited a sharp increase in membrane damage post-treatment, which peaked at a midpoint and then plateaued or slightly declined, suggesting a strain-specific or concentration-dependent effect. These findings confirm the antibacterial efficacy of AgNPs in disrupting bacterial membranes in a manner that is dependent on both concentration and bacterial strain. The differences in response among bacterial strains highlight the selective activity of AgNPs, suggesting their potential for targeting specific bacterial pathogens.

## Discussion

The biosynthesis of silver nanoparticles (AgNPs) using yeast isolates from Sudanese soil presents an eco-friendly and sustainable alternative to conventional chemical synthesis. Unlike methods reliant on toxic reagents and energy-intensive processes, yeast-mediated synthesis leverages biological reduction mechanisms, aligning with green nanotechnology principles. While single-celled yeasts remain underexplored in AgNP biosynthesis, prior studies have reported *Candida albicans* and *Candida lusitaniae* as effective producers [11]. This study introduces *C. parapsilosis* as a promising candidate for AgNP synthesis, demonstrating notable antimicrobial efficacy.

AgNP formation was visually confirmed by the characteristic brown color change, attributed to surface plasmon resonance (SPR), indicating Ag⁺ reduction and electron excitation under electromagnetic radiation. Similar SPR-based confirmations have been reported in fungal-mediated synthesis [24]. Key reaction parameters—AgNO₃ concentration, incubation time, and temperature—were optimized, revealing that a 24-hour incubation at 25 °C in darkness was sufficient for complete nanoparticle formation. AgNP biosynthesis likely involves Ag⁺ interaction with negatively charged functional groups on the yeast cell wall, followed by electrostatic attraction and enzymatic reduction via nitrate and sulfate reductases, utilizing ATP and NADH. Intracellularly, Ag⁺ binds to phytochelatins, forming Ag-PC complexes that degrade within vacuoles, releasing AgNPs [26].

Optimal AgNO₃ concentrations yielded monodisperse AgNPs (5.7–6.5 nm), whereas higher precursor concentrations produced larger, polydisperse particles. The SPR band at 419 nm confirmed particle size. Compared to previous studies, such as one in Saudi Arabia where fungi synthesized AgNPs (10–16 nm) with lower antimicrobial activity [19], our yeast-derived AgNPs exhibited superior antibacterial efficacy against *E. coli* and *S. aureus*. These variations may stem from differences in yeast species, reaction conditions, or nanoparticle stability [24, 26].

Characterization via UV-Vis spectroscopy, X-ray diffraction (XRD), transmission electron microscopy (TEM), high-resolution TEM (HRTEM), and selected area electron diffraction (SAED) confirmed the crystalline nature and morphology of AgNPs. SPR peaks (400–450 nm) were consistent with prior reports [27], and XRD peaks at 37.8°, 45.9°, 65.1°, and 77.01° corresponded to the (111), (200), (220), and (311) planes of silver. Slight shifts in peak positions suggest synthesis-dependent structural variations. TEM and HRTEM confirmed spherical morphology, with lower AgNO₃ concentrations yielding smaller, well-dispersed nanoparticles. The lattice fringes observed (0.24 nm interplanar spacing) aligned with the (111) plane of metallic silver. SAED patterns displayed bright concentric rings, confirming a polycrystalline nature [29, 30].

Yeast-synthesized AgNPs exhibited strong antibacterial activity, particularly against *S. aureus*, with a 28 mm inhibition zone and 23.8 mm inhibition zone for *E.coli*. Compared to other studies, such as Thaer et al. (2023) reporting 18.5 mm (*E. coli*) and 12 mm (*S. aureus*) [31], and Sagar and Ashok (2012) with 16 mm (*E. coli*) and 13 mm (*S. aureus*) [32], our findings indicate enhanced efficacy. While chemical synthesis by Attia et al. (2017) achieved a 26.5 mm zone for *E. coli* [33], our yeast-mediated approach offers superior biocompatibility and sustainability.

The antimicrobial efficacy of silver nanoparticles (AgNPs) synthesized from Sudanese *Candida parapsilosis* was compared with AgNPs derived from other sources, as summarized in Table 5. The results demonstrate that the AgNPs produced in this study exhibit superior inhibitory effects against both Gram-positive and Gram-negative bacteria, including multidrug-resistant (MDR) clinical isolates, compared to AgNPs synthesized from other fungal or chemical sources. As shown in Table 5, the inhibition zones of Sudanese *C. parapsilosis*-derived AgNPs are larger than those reported in studies using fungi from Saudi Arabia, India, and the Himalayas. This highlights the potential of yeast-mediated AgNP synthesis as a sustainable and effective antimicrobial approach. The differences in inhibition may be attributed to variations in nanoparticle size, synthesis conditions, and bacterial susceptibility.

**Table 5:** Comparative analysis of the inhibitory effects of AgNPs synthesized from Sudanese *C. parapsilosis* and other origins

Source of AgNPs	Bacterial Strain	Synthesis Method	Nanoparticle Size (nm)	Concentration (mg/mL)	Inhibition Zone (mm)	Reference
*C. parapsilosis* (Sudan)	*E.coli* (ATCC 43890)	Yeast-mediated biosynthesis	5.7–6.5	250 mg/mL	24 mm	Current Study
	*S. aureus (MTCC 96)*			250 mg/mL	28 mm	Current Study
	*S. typhi (Clinical Isolate)*			250 mg/mL	24.5 mm	Current Study
Fungi (Saudi Arabia)	*E. coli*	Fungi-mediated biosynthesis	12–20	Not specified	18.5 mm	Thaer et al. (2023)
	*S. aureus*			Not specified	12 mm	
Soil Fungi (Himalayan)	*E.coli*	Fungi-mediated biosynthesis	10–16	Not specified	16 mm	Devi and Joshi (2012)
	*S. aureus*			Not specified	13 mm	
*Azadirachta indica* (India)	*S. typhi*	Plant-mediated synthesis	20–30	Not specified	21 mm	Amesh et al., 2015
Marine Endophytic Fungi (*Cladosporium cladosporioides*) (India)	*E.coli*	Fungi-mediated biosynthesis		Not specified	18 mm	Hulikere and Joshi (2019)
	*S. aureus*			Not specified	15 mm	
Chemical Synthesis (Egypt)	*E.coli*		10–20	Not specified	26.5 mm	Attia etal. (2017) [34]/ Attia et al. (2016) [35]

The antimicrobial effect of AgNPs was concentration-dependent, with the highest efficacy at 250 mg/mL, resulting in an average inhibition zone of 27.3 ± 2.2 mm. *P. aeruginosa* exhibited the greatest susceptibility (29 mm), likely due to its efflux pumps that actively expel both AgNPs and antibiotics. At 500 mg/mL, inhibition zones slightly decreased, possibly due to nanoparticle aggregation reducing bioavailability, a phenomenon previously reported [36]. Among clinical isolates, *S. typhi* (24.5 ± 0.58 mm) and *E. coli* (23.8 ± 0.79 mm) showed the strongest inhibition, suggesting that AgNPs enhance antibiotic uptake by disrupting bacterial membranes.

The antimicrobial efficacy of AgNPs varies based on bacterial cell wall structure, size, surface area, and morphology [37]. Gram-negative bacteria, with their thinner peptidoglycan layer, are more susceptible to AgNP-induced damage than Gram-positive bacteria [38

]. Additionally, the crystal structure of AgNPs influences bactericidal activity, with cubic nanoparticles exhibiting superior effects due to their highly reactive surfaces [39].

Beyond their direct antimicrobial action, AgNPs overcome bacterial resistance mechanisms, including efflux pump inhibition, biofilm disruption, and enzyme inactivation. Efflux pumps, such as AcrAB-TolC in *E. coli* and *K. pneumoniae*, reduce intracellular antibiotic concentrations, but AgNPs counteract this by disrupting the proton motive force and binding to efflux proteins, restoring antibiotic efficacy. AgNPs also inhibit biofilm formation by generating reactive oxygen species (ROS) that degrade extracellular matrices, interfering with quorum sensing (QS) molecules, and depolarizing bacterial membranes to enhance antibiotic penetration. Studies have shown that AgNP treatment reduces *P.aeruginosa* biofilm biomass by 83%, significantly enhancing colistin efficacy. Additionally, AgNPs inhibit bacterial resistance enzymes, such as β-lactamases, by chelating zinc (Zn²⁺) or serine residues, while also downregulating resistance gene expression. This inhibition restores the efficacy of β-lactam antibiotics, particularly against extended-spectrum β-lactamase (ESBL)-producing *K. pneumoniae*.

The multi-faceted action of AgNPs—targeting efflux pumps, biofilms, and resistance enzymes—demonstrates their broad-spectrum antimicrobial potential. By disrupting multiple resistance pathways, AgNPs may help mitigate the development of bacterial resistance more effectively than conventional antibiotics. Our findings align with previous studies confirming the strong antimicrobial potential of AgNPs against multidrug-resistant pathogens [38, 40].

Notably, MIC and MBC variability among bacterial strains highlights the role of nanoparticle characteristics in antimicrobial efficacy. Unlike Lotfy et al. [38], who primarily ranked bacterial susceptibility, our study provides a detailed classification of bactericidal versus bacteriostatic effects based on MBC/MIC ratios, emphasizing the influence of bacterial cell wall composition and resistance mechanisms. Differences in MIC and MBC values across studies may arise from nanoparticle size, surface coatings, or bacterial strain variation [41, 42].

The results of this study align with and expand upon previous research demonstrating the synergistic potential of silver nanoparticles (AgNPs) when combined with conventional antibiotics.as reported by Sheik et al. [43] reported an overall percentage increase in the average fold-area of inhibition zones when antibiotics were coated with AgNPs, supporting the concept that AgNPs enhance antibiotic efficacy. Similarly, the findings of Devi and Joshi [44] showed a modest increase in inhibition zones (0.06 to 1.89-fold) when erythromycin, methicillin, chloramphenicol, and ciprofloxacin were combined with fungal-derived AgNPs. However, the current study demonstrates substantially greater fold increases, particularly in *E. faecalis* (up to 9.84-fold with Colistin) and *A. baumannii* (up to 5.11-fold with Ceftazidime), suggesting that AgNP-enhanced antibiotic efficacy varies depending on bacterial species, nanoparticle synthesis method, and antibiotic type.

A key difference between the present study and that of Devi and Joshi [44], lies in the magnitude of synergy observed. While their study documented small to moderate fold increases, our results indicate a more pronounced effect, particularly with Colistin, Ceftazidime, and Ampicillin. This suggests that differences in AgNP size, morphology, and source of synthesis could influence antimicrobial synergy. The stronger enhancement observed in this study may also be due to species-specific bacterial resistance mechanisms, as statistical comparisons revealed significant differences across bacterial groups (*p* < 0.0001).

Additionally, while Devi and Joshi [44] focused primarily on Gram-positive bacteria, the present study demonstrates broad-spectrum synergy across both Gram-positive and Gram-negative MDR pathogens. The enhanced effect in *A. baumannii* and *K. pneumoniae* suggests that AgNPs may disrupt outer membrane permeability, thereby increasing antibiotic uptake, while the significant synergy observed in *S. aureus* and *E. faecalis* supports the hypothesis that AgNPs interfere with cell wall integrity and resistance enzyme function.

This study reinforces prior research on the potent antibacterial effects of green-synthesized silver nanoparticles (AgNPs), particularly in disrupting bacterial membrane integrity and permeability. Consistent with previous studies, our findings demonstrate that 45.8% of bacterial isolates exhibited compromised membrane integrity following AgNP treatment, supporting earlier reports that AgNPs destabilize bacterial membranes, ultimately leading to cell death [16].

While biosynthesized AgNPs offer a sustainable alternative to antibiotic drugs, their long-term environmental impact must be considered. AgNPs can accumulate in soil and water, altering microbial diversity and disrupting nitrogen cycling. Studies suggest AgNP exposure affects beneficial soil bacteria, potentially impacting plant growth and agricultural productivity. In aquatic ecosystems, AgNPs may bioaccumulate in organisms, causing oxidative stress and membrane damage. Although transformation into silver sulfide (Ag₂S) in wastewater reduces toxicity, incomplete removal raises concerns about residual pollution. To mitigate risks, future research should focus on biodegradable AgNP formulations, eco-friendly coatings, and improved disposal methods, alongside long-term ecological assessments to ensure minimal environmental harm [8].

Moreover, membrane permeability was assessed through the hydrolysis of O-nitrophenyl-β-D-galactopyranoside (ONPG), revealing a rise in absorbance at 420 nm, further confirming AgNP-induced membrane disruption. This aligns with prior studies emphasizing the ability of AgNPs to alter bacterial membrane permeability, contributing to their antimicrobial efficacy. Additionally, shifts in zeta potential observed in *S. aureus*, *Shigella dysenteriae*, and *S. typhi* indicate significant alterations in membrane charge and stability, further corroborating previous findings on AgNP-induced membrane perturbations [45]. These results, in agreement with existing literature, highlight AgNPs as a promising eco-friendly alternative to conventional antibiotics. By leveraging their membrane-disrupting capabilities, AgNPs offer a novel and sustainable approach to combating bacterial infections and antibiotic resistance, reinforcing the growing body of evidence supporting their application in antimicrobial strategies [24, 46].

This study underscores the potential of yeast-mediated AgNPs as a sustainable antimicrobial strategy against AMR. However, further optimization of synthesis parameters and in vivo validation are necessary to facilitate clinical translation. Addressing challenges such as cytotoxicity, stability, and large-scale production will be crucial for future applications in medicine and biotechnology.

## Conclusion

This study successfully demonstrated the novel eco-friendly synthesis of silver nanoparticles (AgNPs) using *C.parapsilosis* strains isolated from Sudanese soil, emphasizing the potential of local fungal biodiversity for sustainable nanotechnology applications. The synthesized AgNPs exhibited unique spherical morphology and high crystallinity, contributing to their remarkable antibacterial efficacy against both Gram-positive and Gram-negative bacteria, including multidrug-resistant (MDR) strains. The findings revealed the ability of AgNPs to disrupt bacterial membrane integrity and enhance the effectiveness of conventional antibiotics, highlighting their potential to combat antimicrobial resistance (AMR). This synergistic interaction not only reduces the required antibiotic doses but also mitigates the spread of resistant pathogens. By integrating green nanotechnology and microbiology, the study provides a sustainable approach to addressing AMR, enhancing public health outcomes, and contributing to environmental preservation in in Sudan and globally. Future work should focus on scaling up production, evaluating in vivo applications, and ensuring safety for broader clinical use. This pioneering effort underscores the value of leveraging indigenous microbial resources to tackle global health challenges and paves the way for further advancements in fungal-based nanoparticle synthesis and application.

## Electronic supplementary material

Below is the link to the electronic supplementary material.

Supplementary Material 1

Supplementary Material 2

## Data Availability

The nucleotide sequences obtained from the fungal isolates have been deposited in the GenBank database and assigned unique accession numbers (PQ796639 and PQ796640).
