# Supplementary material for: Green synthesis of silver nanoparticles using Sudanese Candida parapsilosis: a sustainable approach to combat antimicrobial resistance
Source: BMC Microbiol. 2025 May 21;25:312. doi: 10.1186/s12866-025-04038-9 (PMC12093675; doi:10.1186/s12866-025-04038-9)
Supplement: Supplementary file 2 — Supplementary Material 2 [file 12866_2025_4038_MOESM2_ESM.docx]

**
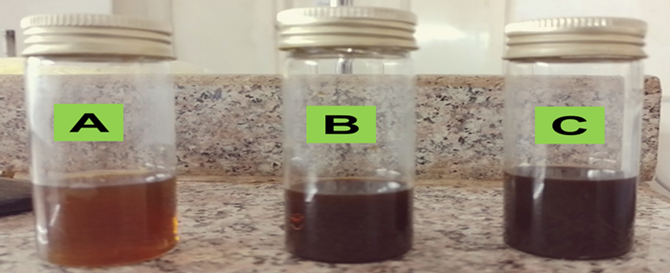
**

**Fig S 1: The color change during the biosynthesis of the AgNPs colloidal solution reflects different concentrations: A (AgNPs-L, low), B (AgNPs-M, medium), and C (AgNPs-H, high)**

**
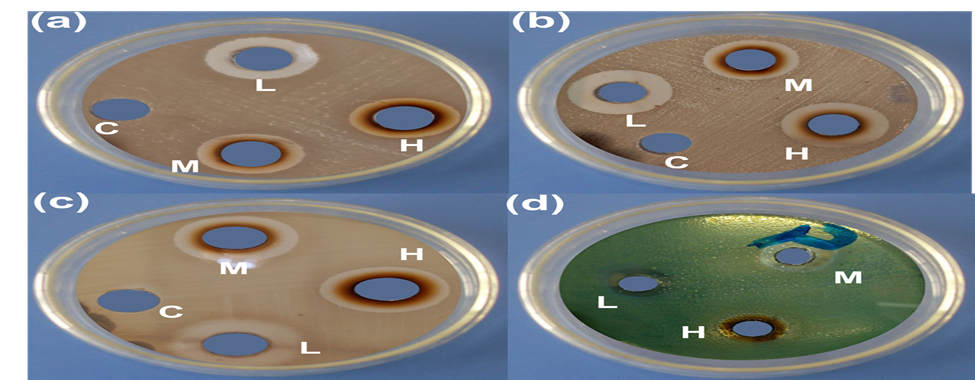
**

**Fig S. 2.** Digital images showing clear inhibition zones for antibacterial activity against (a) *E. coli* (ATCC 43890), (b) *E. faecalis* (MTCC 2729), (c) *S. aureus* (MTCC 96), and (d) *P. aeruginosa* (ATCC). The labels represent C (control), L (low concentration), M (medium concentration), and H (high concentration).

**
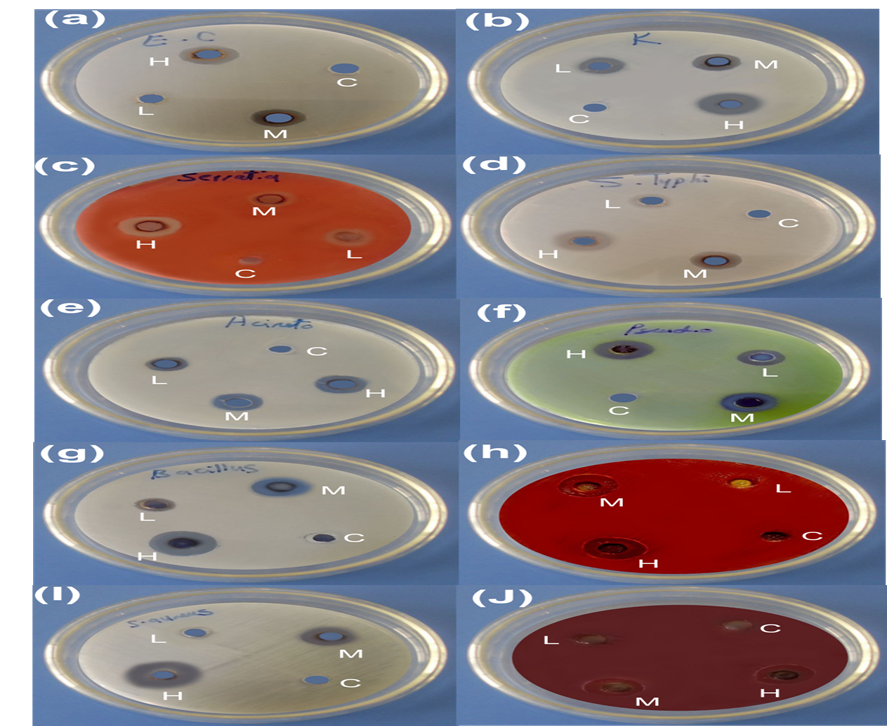
**

**Fig S, 3.** Inhibitory effect of AgNPs against resistant bacterial species: (a) *E. coli*, (b) *K. pneumoniae*, (c) *P. aeruginosa*, (d) *A.baumannii*, (e) *Serratia marcescens*, (f) *S. typhi*, (g) *S. aureus,* (h) *Enterococcus faecalis*, (i) *Listeria monocytogenes*, and (j) *Bacillus cereus*. The discs represent different concentrations of AgNPs: C (control), L (low concentration), M (medium concentration), and H (high concentration).
